# Detection of *GSTM1*-null Genotype in Women Undergoing IVF Treatment

**DOI:** 10.3390/jcm12237269

**Published:** 2023-11-23

**Authors:** Ioannis Chrysanthopoulos, Angelos Petsavas, Despoina Mavrogianni, Anastasios Potiris, Nikolaos Machairiotis, Eirini Drakaki, Dionysios Vrachnis, Pavlos Machairoudias, Theodoros Karampitsakos, Paraskevas Perros, Christos Koratzanis, Peter Drakakis, Sofoklis Stavros

**Affiliations:** 1 First Department of Obstetrics and Gynecology, Alexandra Hospital, Medical School, National and Kapodistrian University of Athens, 115 28 Athens, Greece; johnchrysan@med.uoa.gr (I.C.); angelpet@med.uoa.gr (A.P.); dmavrogianni@med.uoa.gr (D.M.); eirinidrak@med.uoa.gr (E.D.); paris_per@yahoo.gr (P.P.); pdrakakis@med.uoa.gr (P.D.); 2 Third Department of Obstetrics and Gynecology, University General Hospital “ATTIKON”, Medical School, National and Kapodistrian University of Athens, 124 62 Athens, Greece; nmachai@med.uoa.gr (N.M.); pavlosmach@med.uoa.gr (P.M.); theokarampi@med.uoa.gr (T.K.); xkoratzanis@med.uoa.gr (C.K.); sfstavrou@med.uoa.gr (S.S.); 3 Medical School, National and Kapodistrian University of Athens, 115 27 Athens, Greece; dvrachnis@med.uoa.gr

**Keywords:** female infertility, *GSTM1* gene, ART, in vitro fertilization (IVF), oxidative stress

## Abstract

Background: Glutathione S-transferase (GST) M1 belongs to a family of detoxification enzymes and deficiency in enzyme activity is due to a homozygous deletion of the *GSTM1* gene. Several studies reveal a possible correlation between female infertility and *GSTM1* polymorphisms. The aim of this study is to investigate the effect of the *GSTM1*-null polymorphism in female infertility as well as in IVF parameters. Methods: In the study group 125 women were classified as infertile according to WHO and 49 women with at least one successful pregnancy and no miscarriages, as control group. Genomic DNA from blood samples was isolated and PCR amplification was applied to determine the presence of *GSTM1*-null genotype. Results: Data analysis demonstrated a statistically significant higher presence of *GSTM1*-null variant in the infertile group compared to the control group. In a subgroup analysis of the infertile group, the estradiol levels, the number of fertilized oocytes as well as the number and the quality of the cumulus-oocyte complex, were statistically significant higher in women detected with the wildtype of *GSTM1* gene compared to those who had the *GSTM1* null genotype (deletion). Conclusions: Our study results propose a possible involvement of GMST1 in female infertility and may help elucidate possible interactions between the microenvironment of oocytes and the oxidative stress.

## 1. Introduction

Infertility is defined as the inability to conceive after 12 or more months of regular unprotected sexual intercourse [1]. Even though the origin of infertility varies, 40% of its etiology is related to female causes [2,3]. Female infertility may be related to obesity, menstrual disorders, endometriosis, diminished ovarian reserve and tubal occlusion, although several other factors have been validated in contributing to female infertility including oxidative stress [4,5].

Two major types of free radical species have been reported: reactive oxygen species (ROS) and reactive nitrogen species (NOS) which may cause disease and cellular damage [6,7]. In the female reproductive system ROS play either a physiological or a pathological role that affects oocytes, embryos, and their micro- or macro-environment. A wide range of physiological reproductive processes, including corpus luteum activity, ovarian steroidogenesis, oocyte maturation, and luteolysis, are modulated by ROS [8]. Oxidative stress (OS) is related to a shift in the equilibrium that leads to an excess of ROS, as cells cannot scavenge free radical species, or prooxidants, and antioxidants [9,10,11]. Oxidative stress may be related increased DNA fragmentation, cellular apoptosis, damaged germ cells and potential poor fertilization outcomes and higher incidence of miscarriage [3]. An increasing scientific attention is drawn to OS effect in female reproduction and its role in the incidence of abortions, hydatidiform moles, preeclampsia, and congenital abnormalities caused by free radicals. Some evidence has surfaced its involvement in endometriosis and in unexplained infertility [7].

Glutathione S-transferase system 1 (*GSTM1* and *GSTT1*) is encoded by two genes, mu (μ) and theta (θ), which express phase II multifunctional enzymes essential for bioactivation processes and cellular detoxification. As a result, *GSTM1* and *GSTT1* are significant antioxidant enzymes involved in steroidogenesis [12]. Like the other members of the glutathione-S-transferases family, *GSTM1* act also as a hormone binding protein. The corresponding encoding sequence is well recognized for its high polymorphism [13,14,15]. Due to an extended deletion of the gene (approximately 10 kb), a null activity allele is produced, leading to a non-functional protein [4,12,16]. The incidence of *GSTM1* and *GSTT1* polymorphisms vary significantly among ethnicities. Even in the same ethnic population the variation persists among different countries. For instance, for the Caucasian population the incidence of the deleted variant of *GSTM1* and *GSTT1* genes is 31.4% and 28.2% respectively [17]. A case control study from the United Kingdom reported a 48.9% incidence of *GSTM1*-null variant [18]. On the other hand, in the Hellenic population, we have previously reported that the incidence of the null variant is 16.7% [19]. It is proposed, that GST genes play a crucial role in female reproduction as they are detected in high levels in the placenta and the ovarian follicles [20]. Apart from that, *GSTM1*-null polymorphism has been associated with increased risk for endometriosis [18,21] and cervical cancer in reproductive age [17]. Regarding male infertility, we have shown that *GSTM1*-null variant is associated with a two-fold higher risk for infertility [22]. Even though many studies focused on the effects of GSTM polymorphisms on fertility issues, there is only one study from Northern Iran reporting the effect of *GSTM1* polymorphism in infertility and pregnancy rate. To our knowledge, this is the first study that utilizes Caucasian population and reports the effects of *GSTM1*-null variant on assisted reproduction parameters.

The aim of the present study is to investigate whether the presence of *GSTM1*-null polymorphism is associated with an increased risk for infertility. Furthermore, we aim to investigate the impact of the *GSTM1*-null polymorphism in the reproductive hormonal profiles, main embryological outcomes, and clinical outcome parameters, such as quality and number of cumulus-oocyte complex (COC) and number of follicles and fertilized oocytes of women under assisted reproduction techniques.

## 2. Materials and Methods

### 2.1. Study Design, Population and Participant Characteristics

This retrospective study was conducted in the Assisted Reproduction Unit of the First Department of Obstetrics and Gynecology in Athens, Greece, throughout a 7-month period. The participants of the study were 174 women (n = 174), aged 27–46 years old with an average age of 37.3 ± 5.1 years (mean ± SD, min = 27 years; max = 46 years) and median age of 38 years. The study group consisted of 125 infertile women with various factors, who underwent assisted reproduction through either IVF or ICSI. In the control group were included 49 women with at least one successful pregnancy and no history of spontaneous abortion or miscarriage. The inclusion criteria were a regular menstrual cycle of 25 to 35 days, maternal age less than 46 years old and a validated function of both ovaries. The exclusion criteria were polycystic ovarian syndrome (as described by Rotterdam criteria), endocrinological pathology, hydrosalpinx or autoimmune disorders [23].

### 2.2. Ethical Approval

The study was conducted in accordance with the Declaration of Helsinki and approved by the Institutional Review Board of Alexandra General Hospital of Athens with the protocol identifier 662/12.01.2022. A written informed consent was obtained by all the participants prior to the initiation of the study.

### 2.3. DNA Extraction and GSTM1 Genotyping

Peripheral blood samples were collected from all women in EDTA-tubes [24]. Extraction of genomic DNA (gDNA) was performed with the PureLink Genomic DNA kit (K182002, Invitrogen, Waltham, MA, USA), according to the manufacturer’s instructions and samples were stored at 4 °C.

The genotyping of the *GSTM1* gene (wildtype) and *GSTM1*-null genotype, was performed through Polymerase Chain Reaction (PCR) [25], using the following primer pairs: *GSTM1* forward (5′-GAACTCCCTGAAAAGCTAAAGC-3′) and *GSTM1* reverse (5′-GTTGGGCTCAAATATACGGTGG-3′). Each PCR reaction was performed in a total volume of 25 μL, containing 100 ng of DNA, 400 nM of each primer, 1.5 mM MgCl_2_, 100 nM of each deoxynucleotide triphosphate (dNTPs) and 1 unit of Taq DNA polymerase. Reaction mixtures were pre-incubated for 10 min at 94 °C (94 °C for 1 min, 58 °C for 1 min, 72 °C for 1 min) × 35 cycles and 72 °C for 10 min. The amplified DNA was electrophoresed through 3% agarose gels stained with ethidium bromide and visualized under UV light. The expected PCR product was for *GSTM1* gene (wildtype) 219 bp, while no PCR product was detected if the *GSTM1*-null genotype (deletion) was present.

### 2.4. Hormone Assays

In day 3 of menstrual cycle FSH, LH, AMH and prolactin levels were measured by electrochemiluminescence immunoassay (Roche Molecular, Biochemicals, Mannheim, Germany) while estradiol levels were measured on the 5th day of the controlled ovarian stimulation and repeated measurements were performed daily until the day of hCG administration, using a commercially available chemiluminescent Microparticle Immunoassay (CMIA) kit (Abbott Laboratories, Abbott Park, IL, USA).

### 2.5. Controlled Ovarian Stimulation (COS)

COS was conducted following the GnRH agonist protocol. In patients less than 35 years old, a long agonist stimulation protocol was implemented. Ovarian suppression was evaluated by ultrasound and serum E2 levels (≤40 pg/mL) before administration of exogenous gonadotrophin. HCG was administered when the cohort of the follicles were larger than 18 mm and serum estrogen levels were within the expected range according to the number of stimulated follicles. Oocytes were retrieved 36 h after the administration of 10,000 IU hCG. In our control group 16 women were less than 35 years old and in our study group 42 women.

In patients with an age of more than 35 years old at the initiation of the cycle, a short agonist protocol was implemented. Ovarian suppression was evaluated by ultrasound and serum E2 levels (≤40 pg/mL). The initiation of the agonist started on the second day of the cycle and gonadotrophin administration with r-FSH added on day 3 at a starting dose of 200 IU and adapted accordingly to obtain the optimal follicular stimulation. In our control group 33 women were older than 35 years old and in our study group 83 women.

Estradiol (E2) levels were measured daily starting 5 days after the initiation of the stimulation until the day of hCG triggering. The first scan for the evaluation of ovarian stimulation progress was performed on day 7 of the cycle and subsequent scans were performed daily until triggering.

### 2.6. Embryological Processes and Pregnancy

Oocyte retrieval was performed 34–36 h after hCG administration. Following oocyte aspiration, oocytes were incubated in culture medium dishes (Universal IVF Medium, Origio a/s, Malov, Denmark). The evaluation of cumulus-oocyte complex (COC) quality was based on Wood and Wildt study [26].

The oocyte maturation/nuclear maturity is validated by the extrusion of the first polar body [27]. The assessment of fertilization status based on confirming the presence of two pronuclei under the microscope before continuing embryo culture and the pregnancy outcome was followed-up by ultrasonographic visualization of one or more gestational sacs or definitive clinical signs of pregnancy and fetal heartbeat presence [28].

### 2.7. Statistical Analysis

Statistical analysis was performed with Statistics Package for Social Sciences (SPSS), version 15, Minitab 12. For qualitative data was used the chi-square test, x^2^, (Fisher’s exact test) and for non-parametric data Mann-Whitney U was used. A *p*-value less than 0.05 (*p* < 0.05) was regarded as statistically significant. Results are presented as mean ± SD.

## 3. Results

### 3.1. Detection of the Presence/Absence of GSTM1-null Genotype (Deletion) in Infertile Women

**Figure 1:**
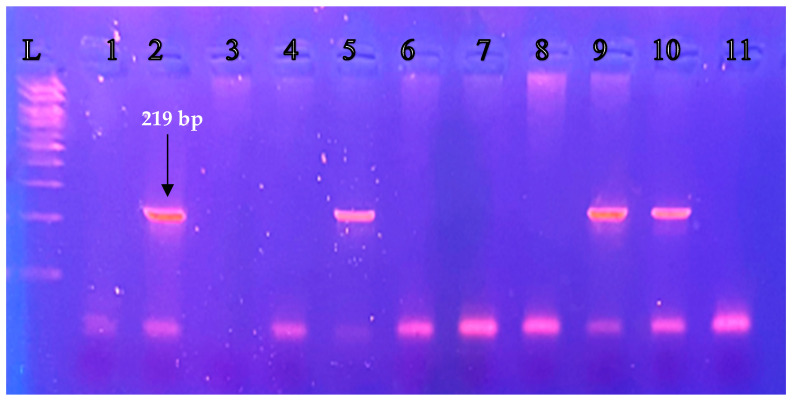
Electrophoresis of PCR products on agarose gel 3% (120 V/45 min) from the study group Lane 2, 5, 9, 10: *GSTM1* gene (wildtype), Lane 1, 3, 4, 6, 7, 8, 11: *GSTM1*-null genotype (deletion), Lane L: DNA ladder (100–1000 bp).

A total of 174 women were included in this study. The study group consisted of 125 infertile women, who underwent IVF treatment and the control group consisted of 49 women. The demographic characteristics of both groups are presented in Table 1. Both groups were matched in ethnicity, age, and body weight. For this study group a fragment size for *GSTM1* gene (wildtype) of 219 bp was expected, while if the *GSTM1*-null genotype (deletion) was present, no PCR product was detected, as shown in Figure 1. From the total of samples investigated, in the infertile group (n = 125), the *GSTM1* gene (wildtype) was detected in 76/125 and the *GSTM1*-null genotype (deletion) was detected in 49/125 samples. In the control group the *GSTM1* (wildtype) was detected in 40/49 and the null variant in 9/49. The probability for having the *GSTM1*-null genotype was statistically significant higher in the infertility group of our study and the *GSTM1*-null genotype was associated with a 2.8-fold increased risk for infertility as it is shown in Table 2. It is important to mention that the incidence of the null variant differ significantly among different populations. Hence, our results reflect the incidence in the Hellenic population and cannot extrapolate in either Caucasians or other ethnicities.

### 3.2. Hormonal and IVF Parameters

In the subgroup analysis (n = 54) comprising from participants of the infertile group with complete medical history. In this new subgroup, we tried to correlate the presence/absence of the *GSTM1*-null genotype with hormonal and IVF parameters. Τhe number of follicles and the number of 2PN embryos in women with the *GSTM1* deletion compared to women with the *GSTM1* wildtype were statistically significant lower (*p* = 0.017 and *p* = 0.013 respectively) as presented in Table 3.

Women with the *GSTM1*-null genotype had a lower number of follicles retrieved compared to women with the wildtype gene, and this result was also statistically significant (*p* = 0.025). The quality of COCs was also significantly lower (*p* = 0.042) in women with the *GSTM1*-null genotype. Furthermore, estradiol (E2) levels on the day of hCG administration in women with *GSTM1*-null genotype were significantly lower (*p* = 0.000) compared to women with the wildtype of *GSTM1* gene. The difference between the number of previous IVF attempts, infertility duration, days of stimulation and oocyte maturation rate was not statistically significant (*p* = 0.484, *p* = 0.510, *p* = 0.300 and *p* = 0.412 respectively) for any of the groups compared. Total FSH administered in both groups was also not statistically significant (*p* = 0.290).

Hormonal profiles (FSH, LH, PRL and AMH) and body mass index (BMI) within patient groups with wildtype *GSTM1* gene or *GSTM1*-null polymorphism, were examined. In the analysis and comparison of hormonal profiles and BMI, no significant difference was demonstrated in the investigated parameters (Table 4) between women who had the *GSTM1*-null polymorphism and women with the wildtype *GSTM1* gene.

### 3.3. Clinical Outcome

**Figure 2:**
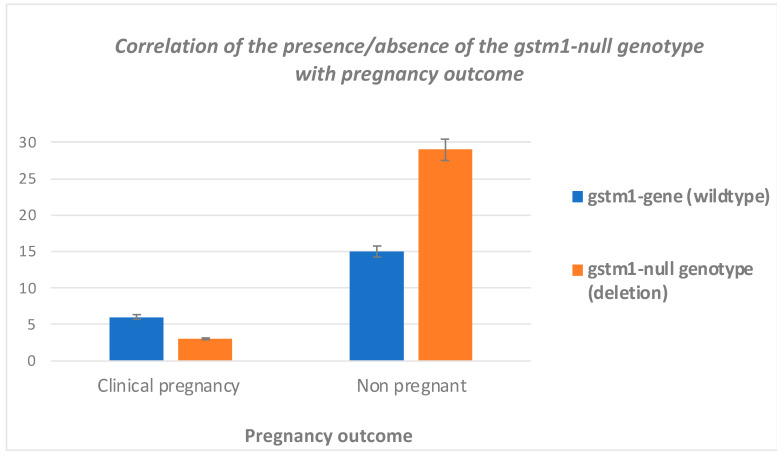
Correlation of the presence/absence of the *GSTM1*-null genotype with pregnancy outcome.

Clinical outcome of the participants was also investigated by the analysis of the data of the participants who achieved a clinical pregnancy with the presence or absence of the *GSTM1*-null genotype (deletion). Nine out of 53 women that underwent IVF had a clinical pregnancy. In the non-pregnant subgroup (44 women), 29 women had the *GSTM1*-null genotype (deletion) and in 15 women the wildtype *GSTM1* gene was present. In the pregnant subgroup, 3 women had the *GSTM1* deletion and 6 had the wildtype *GSTM1* gene (Figure 2). Based on the results obtained through Fisher’s exact test, there is no correlation between the presence or absence of the *GSTM1*-null genotype (deletion) and the pregnancy outcome (*p* = 0.131).

## 4. Discussion

The present study was designed to investigate the possible association between the presence of *GSTM1*-null genotype and infertility in women undergoing IVF treatment. Specifically, it focused on the possible impact of the *GSTM1* polymorphism on different IVF parameters, reproductive hormonal levels, and pregnancy outcome in infertile women, who underwent IVF treatment. This study confirmed that *GSTM1*-null genotype was present in 39% of the infertile women, hence its presence may be related with reproductive pathological conditions and infertility.

Oxidative stress involvement in unexplained infertility and endometriosis has been previously supported [29]. Zhang et al. demonstrated that the *GSTM1*-null genotype is an independent risk factor for the development of endometriosis, and it is involved in primary infertility [13]. Mavrogianni et al. revealed that the absence of *GSTM1* gene detected in women with endometriosis, may indicate a possible involvement of the detoxifying metabolic pathway in the pathophysiology of the disease. Moreover, a recent meta-analysis indicated that the *GSTM1*-null genotype is probably a potential genetic marker for the risk of endometriosis [21].

Many studies have indicated that there is a correlation between *GSTM1* polymorphisms and diseases in both men and women. Bodal et al. demonstrated that polymorphism of both *GSTM1* and *GSTT1* increase the risk of developing breast cancer [30]. Moreover, it has been suggested that *GSTM1*-null genotype is an important genetic risk factor for gastric, lung and colorectal cancer development [31,32]. Another pathology that has been associated with *GSTM1* polymorphisms is glaucoma, with increased incidence of primary open-angle glaucoma (POAG) in the null variant [33]. Similarly, the *GSTM1*-null genotype is linked to an elevated risk of POAG in smokers, according to a study by Stamenkovic et al., suggesting a possible gene-environment interaction [34] and a meta-analysis by Yu et al. demonstrated that combinations of GST polymorphisms are linked to an increased risk of glaucoma [35].

Moreover, the *GSTM1*-null genotype can interact with mercury (Hg) and this interaction seems to play an important role in lower birth weight [36]. The susceptibility to some other environmental heavy metals toxicity, such as lead (Pb) and cadmium (Cd) in blood, is associated with *GSTT1* and *GSTM1* polymorphisms [37]. *GSTM1*- and *GSTT1*-null genotypes have also been associated with male infertility. In Chinese population, a study revealed that *GSTM1*- and *GSTT1*-null genotype contribute to susceptibility to spermatogenesis impairment [38]. A meta-analysis by Li et al. demonstrated that males with dual null genotypes of *GSTM1*/*GSTT1* are particularly susceptible to idiopathic infertility among Caucasians, where the *GSTM1*-null genotype contributes to an elevated risk of male idiopathic infertility [39]. These findings are in accordance with another meta-analysis by Wu et al., who revealed that the risk of *GSTM1* polymorphism was associated with male infertility in both Asian and Caucasian groups [40].

In this study, it has been established that some crucial IVF parameters present a statistically significant difference in women with the *GSTM1* polymorphism, as compared to women with the wildtype gene. Specifically, the presence of the *GSTM1*-null genotype has been linked with lower number of follicles and 2PN oocytes. Additionally, in women with the *GSTM1* polymorphism the number of cumulus-oocyte complexes and especially the number of excellent/good quality cumulus-oocyte complexes was significantly lower compared to women with the wild type *GSTM1* gene. These results indicate that the presence of this specific polymorphism alters in total the result of the ovarian stimulation and the oocyte quality, which can lead to poor quality embryos.

One of the main findings of this study is the difference in the E2 levels on the day of hCG administration. Specifically, E2 levels in women in which the *GSTM1*-null genotype was present, were significantly lower than in women with the wildtype gene. These findings are in accordance with other studies which demonstrated that catechol-O-methyl- transferase (COMT) and glutathione S-transferases (GSTs) have a major role in estrogen synthesis and metabolism and in the extraction of catechol estrogens (CEs) [41]. Estrogens are mainly metabolized by the catechol estrogens pathway (CEMP), which produces ROS and may cause mutations and DNA damage [42]. The CEs are oxidized to catechol estrogen quinones, which can then be conjugated with glutathione by the action of glutathione-S-transferases (GSTs) like *GSTM1* [43]. Because of the *GSTM1*-null genotype, this enzyme is not functional in this estrogen metabolism pathway resulting in lower E2 levels in those women.

Regarding the possible association between the presence of the *GSTM1* polymorphism and the sex hormone levels (FSH, LH, AMH, PRL), this study demonstrated that there is no statistical difference. Saadat et al. reported that among males exposed in chemicals, the only statistically significant difference between men with the *GSTM1*-null genotype, was in testosterone levels. On the contrary, there was no significant difference in FSH and LH levels [44]. In females exposed to natural sour gas with the presence of the *GSTM1* polymorphism, the testosterone levels were also significantly altered [45].

It has been reported that the presence of *GSTM1*-null genotype may influence IVF outcome and increase the risk of recurrent pregnancy loss (RPL). A study conducted in Italian women by Polimanti et al. has demonstrated that GSTA and *GSTM1* variants may play a major role in RPL risk [46]. Nair et al. in their study and meta-analysis reported that there is a significant increased risk of RPL associated with *GSTT1* and *GSTM1* polymorphisms but these findings cannot be considered across different populations, as this association seems to depend on the ethnicity [47]. Another study in northern Iran has demonstrated that there is a significant association between the *GSTM1* polymorphism and IVF outcomes [48]. On the contrary, our study revealed that there is no correlation between the presence or absence of the *GSTM1*-null genotype (deletion) and clinical pregnancy.

Infertility involves molecular and cellular mechanisms, which influence the response of women during the ovarian stimulation. Researchers implicated in Reproductive Biology, but also IVF professionals, are in need of potential biomarkers, which will indicate the possibilities of a succesful fertilization. As already mentioned in our study, oxidative stress influences the oocyte and embryo quality and consequently the fertilization rates. Establishing a possible relation between *GSTM1*, an oxidative stress biomarker, and embryological parameters may play a significant role in the modulation of gamete interaction and mainly in a successful fertilization. For future research directions, it would be of great interest to detect and correlate a possible relation between the presence and absence of *GSTM1* polymorphism and consequently the absence of the enzymatic activity of the encoded protein, with other proteins involved in metabolism like Cytochrome P450 or Glutathione Peroxidase (implicated in the protection of erythrocytes from an oxidative breakdown). Additionally the absence of enzymatic activity should also be studied in relation with inflammatory conditions, as a possible interaction between the Glutathione transferase and the cytokines may be revealed.

To our knowledge, our study is among the first that report the incidence and the effects of *GSTM1* polymorphisms in infertile population. Furthermore, we conducted a further analysis exploring the effect of *GSTM1* polymorphisms on hormonal profile, main embryological outcomes, and clinical outcome parameters, such as quality and number of cumulus-oocyte complex (COC) and number of follicles and fertilized oocytes of women under assisted reproduction techniques. Our study limitations include the small number of the sample in the subgroup analysis and the high variance of the *GSTM1* polymorphisms across ethnicities. It is important to mention that our results reflect the incidence of *GSTM1* polymorphisms in the Hellenic population and further studies are needed to extrapolate our results to the general population.

## 5. Conclusions

In conclusion, our results suggest that the presence of *GSTM1*-null genotype is associated with almost a 3-fold higher risk for infertility in women. Moreover, the presence of the *GSTM1*-null genotype has a significant association with specific IVF parameters, such as the number of follicles, 2PN embryos, COC and COC quality. It is also significantly associated with the E2 levels on the day of hCG administration. Moreover, we observed no correlation between the *GSTM1* polymorphism and clinical pregnancy outcome in women undergoing IVF treatment. As a surrogate marker of infertility, *GSTM1*-null genotype presents a promising result through the reported correlation and further data should validate its importance in the investigation of female factor infertility.

## Figures and Tables

**Table 1:** Baseline characteristics of control (n = 49) and study (n = 125) group.

	Control Group	Infertility Group
Sample size	49	125
Ethnicity (Caucasian)	49	125
Age (in years)	37.10 ± 4.67	36.20 ± 5.24
Weight (in kilograms)	59.2 ± 6	60.9 ± 9
BMI (kg/m^2^)	23.7 ± 1.8	22.87 ± 3.22
Infertility duration (in years)	n/a	4.20 ± 2.5

Values are presented as means ± standard deviation. BMI: Body Mass Index, n/a: not applicable.

**Table 2:** Frequencies of *GSTM1*-null genotype in control (n = 49) and study (n = 125) groups.

		Group		Risk Estimate		
		Control	Infertile	OR	95% CI for OR	*p*-Value
*GSTM1*-null type		9 (18.37%)	49 (39.20%)	2.865	(1.278 6.424)	0.0087
*GSTM1*-wild type		40 (81.63%)	76 (60.80%)			

Pearson’s Chi-square test is used.

**Table 3:** Comparisons of IVF parameters in women of the infertile group carrying the *GSTM1*-null genotype (deletion) versus women with the wildtype of *GSTM1* gene.

Variables	*GSTM1*-null Genotype (Deletion)	N	Mean	SD	*p*-Value (5%)
Infertility Duration	Presence	32	4.50	2.83	0.510
	Absence	22	3.82	2.32	
Days of Stimulation	Presence	32	9.78	1.26	0.300
	Absence	20	9.30	1.26	
Number of Follicles	Presence	32	6.13	3.09	0.017 *
	Absence	21	8.29	2.99	
Number of COC	Presence	32	5.45	3.03	0.025 *
	Absence	22	7.36	3.23	
Number of excellent/good quality COC	Presence	32	4.13	2.57	0.042 *
	Absence	21	5.43	2.29	
% Maturation Rate	Presence	32	71%	20%	0.412
	Absence	21	69%	12%	
Number of 2PN embryos	Presence	32	4.13	2.21	0.013 *
	Absence	21	5.57	2.40	
Number of previous IVF cycles	Presence	32	1.79	1.05	0.484
	Absence	22	1.50	0.74	
Total FSH administered (IU)	Presence	32	3060.94	1170.42	0.920
	Absence	21	2984.52	893.19	
E2 (pg/mL)	Presence	32	1460	808	0.000 *
	Absence	20	2512	1007	

* Means a statistically significant association.

**Table 4:** Comparisons of hormonal levels and BMI in women of the infertile group carrying the *GSTM1*-null genotype (deletion) versus women with the wildtype of *GSTM1* gene.

Variables	*GSTM1*-Null Genotype (Deletion)	N	Mean	SD	*p*-Value (5%)
FSH (mIU/L)	Presence	32	10	14	0.729
	Absence	21	9	5	
LH (mIU/L)	Presence	32	6.08	6.97	0.126
	Absence	22	6.60	3.64	
PRL (ng/mL)	Presence	28	12.97	6.61	0.194
	Absence	17	15.36	7.04	
AMH (pg/L)	Presence	32	9.56	8.06	0.232
	Absence	22	13.95	11.69	
BMI (kg/m^2^)	Presence	32	23.25	3.10	0.519
	Absence	22	22.79	3.52	

## Data Availability

Data are contained within the article.
